# Editorial: Chronic Suppurative Lung Disease and Bronchiectasis in Children and Adolescents

**DOI:** 10.3389/fped.2017.00196

**Published:** 2017-09-05

**Authors:** Kah Peng Eg, Virginia Mirra, Anne B. Chang, Francesca Santamaria

**Affiliations:** ^1^Department of Respiratory and Sleep Medicine, Lady Cilento Children’s Hospital, Children’s Centre for Health Research, Queensland University of Technology, Brisbane, QLD, Australia; ^2^Department of Pediatrics, University of Malaya, Kuala Lumpur, Malaysia; ^3^Department of Translational Medical Sciences, Federico II University, Naples, Italy; ^4^Child Health Division, Menzies School of Health Research, Charles Darwin University, NT, Australia

**Keywords:** COPD, bronchiectasis, lung function, chronic suppurative lung disease, cystic fibrosis, bacterial bronchitis

**Editorial on the Research Topic**

**Chronic Suppurative Lung Disease and Bronchiectasis in Children and Adolescents**

Protracted bacterial bronchitis (PBB) chronic suppurative lung disease (CSLD) and bronchiectasis unrelated to cystic fibrosis (CF) are commonly seen in pediatric specialist respiratory clinics (1–3). Chronic wet cough is common to these conditions and many groups (4, 5) have demonstrated infection and intense inflammation in the lower airways of these children. The importance of these conditions includes:

- These conditions cause respiratory morbidity in developed and developing countries, with some affluent countries reporting childhood fatalities (6). In many centers, these conditions are increasingly diagnosed (7–9) for a variety of reasons, including improved recognition.
- These conditions can be effectively treated leading to improved quality of life (Quell), and clinical outcomes, through reducing both short- and long-term morbidity (1, 10). The longer the chronic wet cough duration when first diagnosed, the worse the radiographic scores in children (4) and adults (11). In a study involving adult-diagnosed broncheictasis, those with chronic cough since childhood had significantly worse disease compared to those without childhood symptoms (e.g., more exacerbations, lower lung function) (11).
- When not optimally managed, accelerated lung function decline and premature death may occur (6, 12, 13). Thus, pediatricians have a crucial role in the early detection and optimal management of these conditions.
- There is increasing evidence that intensive treatment of children who either have CSLD, or who are at risk of developing severe CSLD, prevents poor lung function in adulthood (14, 15). Cohort data found that, ~80% newly diagnosed adults with broncheictasis were symptomatic since childhood, and duration of chronic cough is related (*r* = −0.51, *p* < 0.001 in non-smokers) to lung function at diagnosis (11) Thus, arguably some of the severe end of this spectrum of illness is preventable or modifiable if treated early (16).

Despite the above, children and adolescents with these conditions receive disproportionately fewer resources (clinical and research), when compared with other chronic lung diseases. Indeed, bronchiectasis is regarded by the European Respiratory Society as one of the most neglected lung disease (17). Thus, Frontiers in Pediatrics has a dedicated research topic (edited by Santamaria and Chang). The eight mini reviews in this series aim to summarize current knowledge, predominantly for clinicians. The articles addressed various aspects, including definitions (Redding and Carter, Ishak and Everard), epidemiology and etiological contributions (McCallum and Binks), one of the major causes [primary ciliary dyskinesia (PCD) (Mirra et al.)], impact on QoL (Nathan et al.), and immunology and microbiology (Pizzutto et al.). On the management arm, the articles on the airway clearance (Lee et al.) and prevention and treatment of exacerbations (O’Grady and Grimwood) provide further in-depth insights in addition to recent reviews (8, 18).

There remains a lack of complete agreement on the definitions of PBB, CSLD, and bronchiectasis, all with chronic endobronchial suppuration. In older literature, as highlighted by Redding and Carter, common suppurative lung conditions include bronchiectasis, empyema, lung abscess, and necrotizing pneumonia. In our current era, the latter three are usually considered different entities. Until recently, bronchiectasis was the most commonly recognized form of lung suppuration in children. However, PBB, now recognized as a distinct pediatric condition (19), is more common than bronchiectasis (Redding and Carter).

In the modern era, the global burden of childhood CSLD and bronchiectasis is still poorly addressed. McCallum and Binks focused on the epidemiology of CSLD and bronchiectasis in children and adolescents. The review provided in-depth data on the prevalence of CSLD and bronchiectasis, finding the wide variability among countries, with peaks among socially disadvantaged indigenous populations of New Zealand, Australia, Alaska, and Canada, and lower prevalence in Europe (McCallum and Binks). While CSLD and bronchiectasis are more common in low- and middle-income countries, it is not uncommon as generally perceived in high income countries (2, 20) where many centers have hundreds of patients with bronchiectasis. Indeed, it is often unappreciated that worldwide, bronchiectasis unrelated to CF is far more common than CF.

Chronic suppurative lung disease and bronchiectasis are associated with several underlying conditions (e.g., PCD, CF, immunodeficiency, post-infectious pneumonia) that may share common clinical features (21) although the prevalence of the common clinical symptoms and signs vary among studies (McCallum and Binks). The main reason for this is likely related to severity when assessed and the underlying cause of the CSLD/bronchiectasis. Nevertheless in most settings, it is likely that PCD is an under diagnosed cause of bronchiectasis. This and recent advances in this field in the diagnosis were highlighted by Mirra et al.. While genetic testing for PCD is far from optimum at this point, undoubtedly this field will progress.

There is little doubt that CSLD and bronchiectasis are an important cause of respiratory morbidity and reduced QoL (Nathan et al.). In affected children and adolescents, a long-term follow-up is mandatory, in order to monitor the progression of lung impairment. Indeed, an often unmet need in children with CSLD and bronchiectasis is a more global approach to the disease, which would hopefully take into account also other relevant issues that are often neglected such as growth, nutrition, and psychological aspects. Considerable stress, anxiety, and depression have been reported in primary caregivers who look after these patients, and economic costs due to the frequent need for health care resources utilization are increasingly appreciated. Nathan et al. reviewed studies on the impact on QoL related to interventions, exacerbations, hospitalizations, antibiotic requirement, and health promotion strategies.

Ishak and Everard outlined several old studies linking chronic wet cough to bronchiectasis and pre-bronchiectasis states. They also described the importance of eliminating on-going lower airway infection and inflammation. Different approaches to the definition of PBB was raised (Ishak and Everard), although a recent approach (1) and consensus on PBB was achieved by the American College of Chest Physicians (22) and also by the European Respiratory Society.

Dysregulated host immune response and persistent microbial infection are the key components resulting in the development of bronchiectasis but its pathophysiology remains poorly defined (8). Pizzutto et al. reviewed immunological concepts, highlighting the importance of the various components of the host response, and the roles of important pathogens focusing on the children with no known underlying disorder. They also explored the challenges and limitations in identifying the causative pathogens and described the scarce data on viruses and new findings on the microbiome (Pizzutto et al.).

On the management front, O’Grady and Grimwood highlighted the importance of preventing exacerbation and reduction of its severity as goals in the management. Achieving this maintains lung health and enhances QoL (O’Grady and Grimwood). One of the most important intervention options to achieve this is utilizing airway-clearance techniques (ACT) (18), reviewed by Lee et al. In the consideration of the large patient variability (e.g. severity of bronchiectasis, age, preference, ability, etc.) plus the many available types of ACT and aids (Lee et al.), it is in the child’s best interest that specialized chest physiotherapists’ regular review occurs as part of the patient’s management plan. Lee et al. provided an overview of these various techniques, a guide to age-appropriate ACT, as no single superior technique.

Some statements within the articles in this research topic are controversial in a field that is now advancing faster than it has ever been, albeit still relatively slowly. Although other mini reviews of childhood CSLD and bronchiectasis could have been included, we hope that this series of articles enhance clinical and research interest and awareness in the field of pediatric CSLD and bronchiectasis. In doing so, health practitioners who see children may improve the lives of many through the early detection, treatment, and consequently likely reducing the long-term detrimental effects of CSLD and bronchiectasis. Large-scale research in this field is still scarce. Extrapolation of therapeutic concepts from the adult studies may not be justified in view of the wide spectrum of the disease.

## Author Contributions

All authors listed have made substantial, direct, and intellectual contribution to the work and approved it for publication.

## Conflict of Interest Statement

The authors declare that the research was conducted in the absence of any commercial or financial relationships that could be construed as a potential conflict of interest.
